# T Cell Responses against Orthopoxviruses in HIV-Positive Patients

**DOI:** 10.3390/vaccines12020131

**Published:** 2024-01-27

**Authors:** Sammet Stefanie, Michael Koldehoff, Pia Schenk-Westkamp, Peter A. Horn, Stefan Esser, Monika Lindemann

**Affiliations:** 1 Department of Dermatology, University Hospital Essen, University Duisburg-Essen, 45147 Essen, Germany; stefanie.sammet@uk-essen.de (S.S.); pia.schenkwestkamp@uk-essen.de (P.S.-W.); stefan.esser@uk-essen.de (S.E.); 2 Zotz Klimas, MVZ Düsseldorf, 40210 Düsseldorf, Germany; koldehoff@zotzklimas.de; 3 Department of Hygiene and Environmental Medicine, University Hospital Essen, University Duisburg-Essen, 45147 Essen, Germany; 4 Department of Hematology and Stem Cell Transplantation, University Hospital Essen, University Duisburg-Essen, 45147 Essen, Germany; 5 Institute for Transfusion Medicine, University Hospital Essen, University Duisburg-Essen, 45147 Essen, Germany; peter.horn@uk-essen.de; 6 Institute for Translational HIV Research, University Hospital Essen, University Duisburg-Essen, 45147 Essen, Germany

**Keywords:** mpox, vaccination, infection, HIV, ELISpot, interferon-gamma, interleukin-2

## Abstract

A global outbreak of predominantly sexually transmitted mpox infections, outside endemic regions, was reported in May 2022. Thereafter, risk groups were vaccinated against smallpox, a structurally related orthopoxvirus. In the current study, we analyzed T cell responses against peptides derived from orthopoxviruses in 33 HIV-positive patients after two vaccinations against smallpox and in 10 patients after mpox infection. We established an ELISpot assay, detecting either the secretion of the pro-inflammatory cytokine interferon (IFN)-γ or interleukin (IL)-2. After vaccination, 21 out of 33 patients (64%) showed specific IFN-γ secretion and 18 (55%) specific IL-2 secretion, defined as >3-fold higher specific value than negative control and at least 4 spots above the negative control. After mpox infection, all patients showed specific IFN-γ secretion and 7 out of 10 (70%) IL-2 secretion. In vaccinated patients, IFN-γ responses were significantly lower than in patients with mpox infection (median response 4.5 vs. 21.0 spots, *p* < 0.001). The same trend was observed for IL-2 responses. After mpox infection, IL-2 ELISpot results positively correlated with CD8+ T cells (*p* < 0.05). Thus, T cell responses were detectable in two thirds of HIV-positive patients after vaccination and were even more abundant and vigorous after mpox infection.

## 1. Introduction

A global outbreak of mpox (termed “monkeypox” until recently) was reported in May 2022, and men between 31 and 35 years of age are, to date, the largest patient group [1]. Mpox was formerly known as viral zoonosis (a virus transmitted from animals to humans) caused by an enveloped double-stranded DNA virus that belongs to the orthopoxvirus genus of the Poxviridae family, which was endemic in Central and West African countries [2]. Mpox can also spread from person to person through broken skin or mucosal surfaces (e.g., oral, genital, anorectal, ocular, and pharyngeal) [2]. Infection causes symptoms similar to those seen in the past in smallpox patients, although it is clinically less severe [2]. The most frequently described symptoms comprise rash, fever, malaise, chills, pruritus, headache, enlarged lymph nodes, myalgia, and rectal pain [1]. On 17 November 2023, the Centers for Disease Control and Prevention globally confirmed more than 91,000 cases [1]. More than 31,000 of these cases were reported from the U.S. [1]. The majority of European cases were found in Spain, France, the United Kingdom, and Germany [3]. Since June 2022, risk groups were vaccinated using vaccines that were proven effective also against smallpox [1,3,4]. Current vaccines contain the live, attenuated vaccinia virus strain Ankara (e.g., Jynneos^®^/Imvanex, Bavarian Nordic A/S, Kvistgaard, Denmark) [4]. The vaccine Imvanex was approved by the U.S. FDA to protect against mpox, and it is considered safe also for immunocompromised individuals such as HIV-positive patients [5,6,7,8]. It is noteworthy that the vaccination of children against the smallpox virus has not been carried out since 1980 [9] when the WHO announced the eradication of the smallpox virus. It is estimated that over 70% of the world’s population is no longer protected against smallpox and, through cross-immunity, against the closely related mpox virus [10]. To date, there is no vaccine specifically directed against mpox, neither a traditional vaccine nor a next-generation vaccine, e.g., based on the messenger RNA (mRNA) technology [11].

It was our aim to establish an ELISpot assay to detect cellular responses against orthopoxviruses (including mpox and smallpox), which was then used to assess cellular in vitro responses after vaccination and infection with the mpox virus in HIV-positive patients. We here assessed the frequency of virus-specific T cells, secreting either the pro-inflammatory cytokine interferon (IFN)-γ or interleukin (IL)-2, and compared HIV-positive patients after vaccination and infection. Furthermore, we evaluated if age, distance to vaccination or infection, and T cell counts had an impact on the strength of T cell responses.

## 2. Materials and Methods

### 2.1. Volunteers

For the establishment and validation of the ELISpot assay, we tested 26 healthy controls (median age 39 years, range 21–68, 8 men, and 18 women). Eleven were vaccinated once and one twice against smallpox in early childhood. Moreover, four healthy volunteers were tested after a recent single shot of vaccination (median interval between vaccination and the first testing 71 days, range 59–88). Three of the vaccinated controls were sequentially tested. Ten healthy controls were unvaccinated and uninfected.

The patient cohort included 43 male, HIV-positive patients with a median age of 44 years (range 24–63), and a median CD4+ T cell count of 768/μL (range 335–1770) (Table 1). Thirty-three patients were tested after vaccination against (m)pox and ten patients after mpox infection. None of the patients had a progress to AIDS within six months prior to testing or thereafter. The groups with vaccination and infection had a similar age (median of 44 v. 39 years). All but two patients had received two recent vaccinations with Imvanex (Jynneos^®^, Bavarian Nordic A/S, Kvistgaard, Denmark). The other two patients were vaccinated once in early childhood and boosted once recently with Imvanex. Cellular immune responses were analyzed at a median of 115 days after the second dose of vaccination or at a median of 314 days after mpox infection.

This study was conducted according to the guidelines of the Declaration of Helsinki and approved by the Ethics Committee of the University Hospital Essen, Germany (SCABIO-HIV). Informed consent was obtained from all subjects involved in this study.

### 2.2. ELISpot Assay

To assess orthopoxvirus-specific cellular immunity, we performed ELISpot assays using a mix of 127 peptides covering selected proteins of the mpox virus, smallpox (variola) virus, and vaccinia virus (PepMix Pan-Poxviridae Select, jpt, Berlin, Germany). All assays included negative and positive controls, i.e., peripheral blood mononuclear cells (PBMCs) left unstimulated or stimulated with the T cell mitogen phytohemagglutinin (1 μg/mL). To optimize the ELISpot conditions, we varied PBMC numbers from 200,000 to 400,000 and used various detection systems (T-Track^®^ ELISpot kit, Mikrogen GmbH, Neuried, Germany; formerly Lophius Biosciences [12] or an in house ELISpot for IFN-γ and IL-2 [13,14]) and different antigen concentrations. Cell cultures were performed for 19–24 h, and resultant cytokine spots were detected by an ELISpot plate reader (AID Fluorospot, Autoimmun Diagnostika GmbH, Strassberg, Germany). Mean values of duplicate cell cultures were considered.

The comparison of various cell numbers per culture showed that 300,000 PBMCs were optimal because it yielded the best discrimination between negative controls and orthopoxvirus-specific responses (Figure S1a). When 200,000 PBMCs per culture were used, the specific responses were too low, and when 400,000 PBMCs were used, the negative controls considerably increased (up to 6 spots). As compared with a T-Track^®^ ELISpot kit (for IFN-γ), which was used for the initial titration of cell numbers, the in house ELISpot for IFN-γ showed 2-fold higher orthopoxvirus-specific responses (*p* = 0.09) (Figure S1b) and was thus used for the subsequent analyses. After initially stimulating the PBMC with 1 μg/mL per peptide, we then used the double-peptide concentration in parallel. We found similar results in the IFN-γ ELISpot (1 vs. 2 μg/mL per peptide) and slightly higher results for 1 μg/mL in the IL-2 ELISpot (Figure S1c). The conditions defined as optimal (300,000 PBMCs per cell culture, in house ELISpot assays, 1 μg/mL per peptide) were considered for further analyses, if not other specified.

As a result, we determined orthopoxvirus-specific responses as spots increment, defined as stimulated minus unstimulated values. Stimulated spot numbers >3-fold higher than negative (unstimulated) controls combined with an increment value of at least 4 were considered positive, based on a previous study, where the detection system was almost identical [13]. However, in the previous study, 250,000 instead of 300,000 PBMCs per culture were used, and thus the cut-off was slightly adapted (4 spots increment instead of 3). When using 300,000 PBMC in healthy volunteers, the negative controls reached a median value of 0 IFN-γ spots and a mean value of 0.3 (range 0–1.5). For IL-2 spots, the median was 0 and the mean 0.5 (range 0–2). In HIV-positive patients, the median and mean (range) was 0.5 and 1.0 (0–6.5) for IFN-γ spots and 1.0 and 1.5 (0–6.5) for IL-2 spots, respectively.

### 2.3. Statistical Analysis

Statistical analysis was performed with GraphPad Prism 8.4.2.679 (San Diego, CA, USA) and IBM SPSS Statistics version 25 (Armonk, New York, NY, USA) software. For the analysis of numerical variables, we used Spearman correlation and linear regression analysis. To assess the impact of categorical covariates, we used Fisher’s exact test and Mann–Whitney or Kruskal–Wallis test with Dunn’s multiple comparisons test, as appropriate. Two-sided *p* values < 0.05 were considered significant.

## 3. Results

### 3.1. Validation of an Orthopoxvirus-Specific ELISpot Assay in Healthy Volunteers

**Figure 1:**
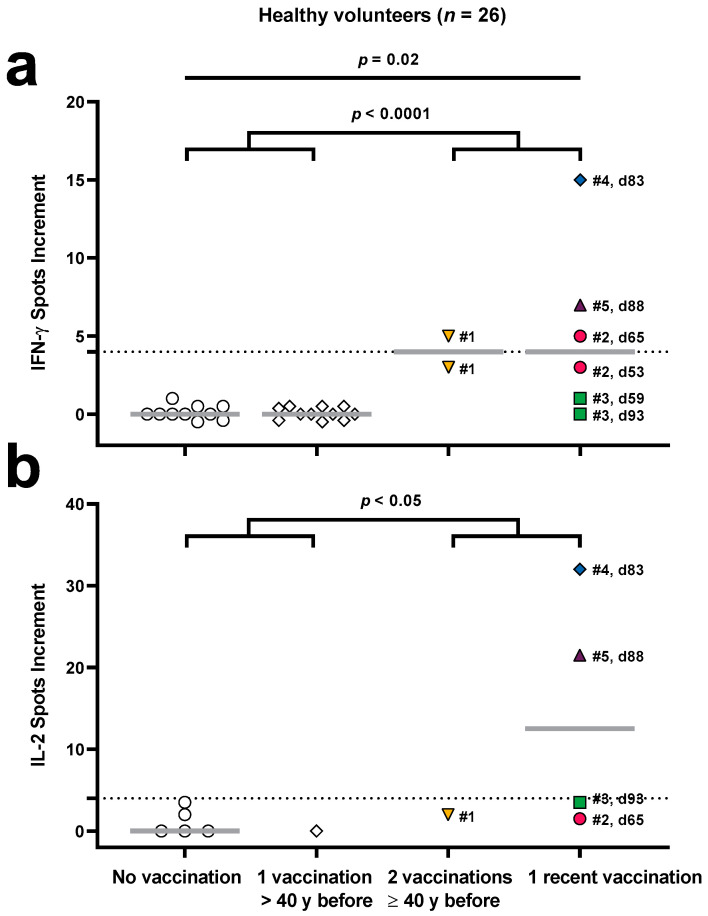
Orthopoxvirus-specific ELISpot assay results in 26 healthy controls. Panel (**a**) shows specific IFN-γ and (**b**) IL-2 spots per 300,000 PBMCs using 1 μg/mL of each orthopoxvirus peptide. Colored symbols indicate controls with either two vaccinations at least 40 years (y) before or with one recent vaccination against smallpox. Each control is shown by an individual color, and the day after one recent vaccination is indicated. Moreover, white symbols indicate controls without previous vaccination against Poxviridae (*n* = 10) or with one vaccination in early childhood, more than 40 years before (*n* = 11). The dotted line shows the cut-off for positive responses (4 spots increment). Four groups were compared by the Kruskal–Wallis test with Dunn’s multiple comparisons test (only for the IFN-γ ELISpot as shown in panel (**a**)) and two groups (with colored or white symbols) by the Mann–Whitney test (panel (**a**,**b**)).

We compared the results of an orthopoxvirus-specific ELISpot assay in 26 healthy controls with or without prior vaccination against Poxviridae (Figure 1). In detail, immunity was borderline in individual #1 with two prior vaccinations (55 and 40 years before). This individual was tested twice with an interval of 47 days, and the resultant spots increment for IFN-γ were 3 and 5 (Figure 1a). At the second time point, we measured in parallel 2 IL-2 spots increment (Figure 1b). In two out of four individuals with a single recent vaccination with the attenuated vaccinia virus strain Ankara (Imvanex), specific IFN-γ and IL-2 responses were detectable (volunteer #4 and #5). Moreover, the IFN-γ response was borderline in volunteer #2. Volunteer #2 was tested twice, at day 53 and day 65 after vaccination, and we could detect 3 and 5 IFN-γ spots increment, respectively. On day 65, we observed 1.5 IL-2 spots increment. In volunteer #3, neither at day 59 nor day 93 after vaccination specific ELISpot responses could be detected. The number of orthopoxvirus-specific IFN-γ spots increment was 15 in volunteer #4 (day 83) and 7 in volunteer #5 (day 88). In parallel, we could detect 32 IL-2 spots increment in volunteer #4 and 21.5 in volunteer #5.

IFN-γ responses were absent in all ten individuals without prior vaccination or infection and in all eleven individuals with one prior vaccination, performed > 40 years before. The results of the IL-2 ELISpot, which was performed in parallel in a subset of samples, were overall similar.

The Kruskal–Wallis test with Dunn’s multiple comparisons test showed that IFN-γ responses in healthy controls without vaccination and with one recent vaccination significantly differed (*p* = 0.02). Moreover, the Mann–Whitney test indicated that IFN-γ responses in healthy controls without vaccination and with only one vaccination in early childhood were significantly lower (*p* < 0.0001) than in controls with two vaccinations in childhood or one recent vaccination (0 vs. 4 spots increment, data represent median values). IL-2 responses in these two groups also significantly differed (*p* < 0.05), with median values of 0 and 3.5 spots increment, respectively.

### 3.2. Orthopoxvirus-Specific ELISpot Results in HIV-Positive Patients after Vaccination and Infection

**Figure 2:**
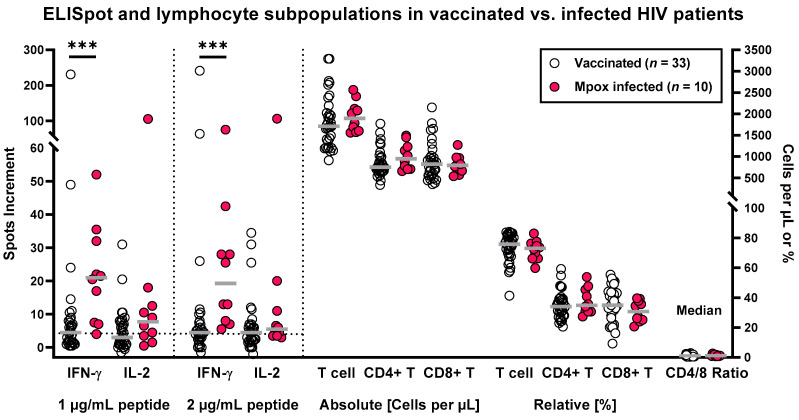
Orthopoxvirus-specific ELISpot assay results and lymphocyte subpopulations in 43 HIV-positive patients. Thirty-three patients were tested after receiving two vaccinations against smallpox and ten after mpox infection. The dotted line (left panel) shows the cut-off for positive responses (4 spots increment). Vaccinated and infected patients were compared by Mann–Whitney test (*** *p* < 0.001).

ELISpot responses toward peptides derived from orthopoxviruses were determined in 33 HIV-positive patients after two vaccinations against smallpox and in 10 patients after mpox infection. Results of the IFN-γ ELISpot were significantly different (*p* < 0.001) between both patient groups irrespective of the peptide concentration used for in vitro stimulation (Figure 2). We obtained median values of 4.5 vs. 21 IFN-γ spots increment when stimulating the cell cultures with 1 μg/mL of each peptide and 4.5 vs. 19 when stimulating with 2 μg/mL. Differences of the IL-2 ELISpot were less pronounced, with median responses of 3 vs. 8 and 4.5 vs. 5.5 IL-2 spots increment in the vaccinated vs. the infected group. These differences in IL-2 secretion were non-significant (*p* = 0.07 for 1 μg/mL of the peptide mix and *p* = 0.13 for 2 μg/mL, respectively). Considering cell cultures after stimulation with 1 μg/mL per peptide, median IFN-γ responses were 4.7-fold higher and IL-2 responses 2.7-fold higher after infection vs. vaccination. Of note, lymphocyte subpopulations did not significantly differ between both patient cohorts (Table 1 and Figure 2).

After vaccination, 19 out of 33 patients (58%) showed specific IFN-γ secretion and 15 (45%) specific IL-2 secretion, defined by an at least 3-fold increase as compared with the negative control and at least 4 spots above the negative control. These numbers are based on stimulation with 1 μg/mL of each peptide. After stimulation with 2 μg/mL of each peptide, we observed slightly higher values. We detected positive IFN-γ responses in 21 out of 33 patients (64%) and IL-2 responses in 18 out of 33 (55%). The patients who displayed specific IL-2 secretion all showed also IFN-γ secretion. Thus, numbers for the combined analysis of both cytokines yield the same result as for IFN-γ responses only.

After mpox infection, all patients (10 out of 10) showed specific IFN-γ secretion irrespective of the peptide concentration. Specific IL-2 secretion was detected in 70% of the patients, which was also independent from the peptide concentration (1 or 2 μg/mL of each peptide). Thus, depending on the cell culture conditions, orthopoxvirus-specific T cell responses were detectable in up to 64% of HIV-positive patients after vaccination and in 100% after mpox infection.

As the ELISpot assay always used 300,000 PBMCs and the percentage of T lymphocytes was very similar in patients with vaccination or mpox infection, the higher ELISpot responses after infection can hardly be explained by differences in lymphocyte subpopulations.

As compared with healthy controls without vaccination against smallpox (Figure 1, unvaccinated, left group), the HIV-positive patients with two vaccinations against smallpox displayed significantly higher orthopoxvirus-specific IFN-γ ELISpot responses (*p* < 0.0001), and the IL-2 ELISpot responses tended to be higher (*p* = 0.08). In the HIV-positive patients with previous mpox infection, IFN-γ and IL-2 ELISpot responses were significantly higher than in these healthy controls (*p* < 0.0001 and *p* = 0.009, respectively).

### 3.3. Correlation of ELISpot Results and Patient Characteristics

Apart from the interval between vaccination or infection and testing, the characteristics of the two patient groups were comparable. As the time to infection was longer, T cell immunity could already have declined during the follow-up. However, our data indicate that T cell responses after infection vs. vaccination were even stronger. Thus, the difference between both groups may have been even more pronounced when testing at a similar interval after contact to Poxviridae.

The Spearman correlation analyses and Mann–Whitney tests were separately performed for patients after vaccination and infection. This analysis included ELISpot results, age, lymphocyte subpopulations, and interval between vaccination/infection and testing.

**Figure 3:**
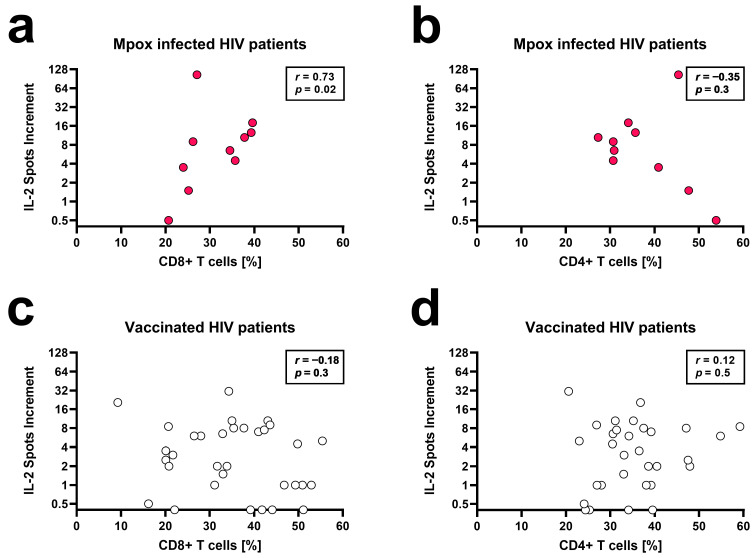
Spearman correlation analysis of orthopoxvirus-specific IL-2 ELISpot results and the percentage of CD8+ T cells and CD4+ T cells in ten patients after mpox infection (**a**,**b**) and in 33 patients after two vaccinations against smallpox (**c**,**d**). Stimulation was performed with 1 μg/mL per peptide.

Although our cohort only comprised ten HIV patients with mpox infection, some of the results already reached statistical significance (Table 2). This is a very specific cohort, so the results are important despite the rather low patient number. Results of the IFN-γ and IL-2 ELISpot correlated positively with relative CD8+ T cell counts (*r* between 0.55 and 0.73) and negatively with the CD4/CD8 ratio (*r* between −0.55 and −0.65). Especially the number of orthopoxvirus-specific IL-2 spots correlated with the percentage of CD8+ T cells (*r* = 0.73, *p* = 0.02, stimulation with 1 μg/mL per peptide) (Figure 3a). Vice versa, the percentage of CD4+ T cells tended to negatively correlate with IL-2 secretion (Figure 3b). After vaccination, ELISpot results and leukocyte subpopulations did not correlate (Figure 3c,d). ELISpot results also did not significantly correlate with age and interval between vaccination or mpox infection and testing. Finally, responses tended to be lower in the three vaccinated patients treated with statins.

## 4. Discussion

The current data indicate that two-thirds of HIV-positive patients can produce IFN-γ and IL-2 secreting orthopoxvirus-specific T cells after two vaccinations against smallpox and all after mpox infection. According to our data, in HIV-positive patients, the infection with mpox induced a stronger T cell immunity than two vaccinations, which has not yet been directly compared in a study. But this finding was expected because after stimulation with our peptide pool, HIV-positive patients with mpox infection are very likely to generate additional T cell responses to mpox epitopes than those only exposed to the vaccine (attenuated variola virus). After infection, orthopoxvirus-specific T cells reached a median frequency of up to 21 IFN-γ and 8 IL-2 secreting cells per 300,000 PBMCs, depending on the cell culture conditions (0.0070% and 0.0027%, respectively). Nevertheless, orthopoxvirus-specific T cells were detectable also after vaccination and reached a median frequency of 4.5 IFN-γ and 4.5 IL-2 secreting cells per 300,000 PBMCs (0.0015%).

IFN-γ responses in HIV-positive patients after two vaccinations were, overall, at a similar level as in healthy adults after a single recent vaccination against Poxviridae. However, IL-2 responses tended to be weaker in vaccinated patients. Our data on healthy controls indicate that one vaccination more than 40 years ago cannot induce a detectable T cell response. Similarly, Matusali et al. described that the majority of healthy individuals vaccinated in childhood, 40 to 60 years ago, did not show specific T cell responses [15]. However, as observed in individual #1, two vaccinations at least 40 years ago may lead to borderline T cell immunity. Although T cell immunity to Poxviridae may fall below a certain threshold and become undetectable after a single smallpox vaccination in early childhood, it has been observed that adults vaccinated in childhood showed milder or even no symptoms after mpox infection, suggesting long-term immunity after vaccination [16,17,18,19]. Moreover, data from an outbreak of mpox infections in Zaire showed a significant clinical benefit of vaccinating against smallpox to control an outbreak of mpox [20]. Between 1980 and 1984, the group from Zaire studied 2510 contacts of 214 patients with mpox infection. Whereas the overall attack rate for contacts without a vaccination scar was 7.2%, it was only 0.9% for those who had been vaccinated in the past.

Of note, another group described stimulation of PBMC by the same peptide pool in seven children aged 0–16 years, exposed to a laboratory-confirmed mpox case [21]. These children had received one vaccination against Poxviridae, with modified vaccinia Ankara–Bavarian Nordic, i.e., a vaccine comparable with the vaccine used in our current study. The children were tested 6 weeks and 15 weeks after this single vaccination using a commercially available system (IFN-γ ELISpot Pro kit, Mabtech, Stockholm, Sweden). ELISpot responses toward the peptide pool were considerably higher in the children as compared with the HIV-positive patients presented in the current study (median of 83 and 70 IFN-γ spots per million PBMCs, i.e., 25 and 21 per 300,000 PBMCs at week 6 and 15, respectively). According to our experience, T cell responses in children are generally higher than in adults, and the HIV infection may additionally have impaired T cell function. Thus, the frequency of orthopoxvirus-specific, IFN-γ secreting cells in the HIV-positive patients fits well with our expectation. In contrast to our study, the study by Ladhani et al. [21] did not measure orthopoxvirus-specific, IL-2 secreting cells. But they determined IL-2 in supernatants of cell cultures infected with mpox virus and found that the concentration of IL-2 vs. IFN-γ was lower, which is in line with a lower frequency of IL-2 vs. IFN-γ secreting cells as determined by ELISpot.

Ladhani et al. [21] showed a balanced CD4+ and CD8+ orthopoxvirus-specific T cell response at both time points after vaccination. Data by Grifoni et al. [22] indicate that vaccination yields memory T cells, with CD4+ responses more frequent and persistent than CD8+ T cell responses. CD4+ and CD8+ orthopoxvirus-specific T cells both produced IFN-γ [22]. Thus, the IFN-γ response we measured after vaccination should also be a response of CD4+ and CD8+ T cells. However, according to our correlation analysis, it can be assumed that after mpox infection, the CD8+ T cells may contribute to a greater extent to the IFN-γ and IL-2 ELISpot response because CD8+ T cells showed significant positive correlation with ELISpot responses in contrast to CD4+ T cells. This assumption fits well with a case report of an HIV-positive woman who showed a dramatic increase in the frequency of CD38+HLA-DR+ CD8+ T cells approximately two months after mpox infection [23]. Similarly, Agrati et al. observed an increase in CD8+ T cells and a concomitant decrease in CD4+ T cells in 16 patients with laboratory-confirmed mpox infection (of which 7 were HIV-positive) as compared with healthy controls [24]. In accordance with the data in humans, animal models suggest that several immune cells contribute to clearance of the mpox virus, with CD8+ T cells playing the main part [25]. Furthermore, Agrati et al. found that almost all patients with mpox infection (15 of 16) developed an orthopoxvirus-specific Th1 response, similar to the current data [24].

Moreover, Agrati et al. reported that T cell responses, determined 8–10 days after symptoms onset, did not seem to be affected by clinical severity or presence of HIV infection [24]. Unfortunately, their data on the IFN-γ ELISpot were not separately shown for patients with and without HIV infection. Estimating the frequency of orthopoxvirus-specific T cells from a figure with a logarithmic scale, it seemed to be only slightly higher than reported in the current study. We analyzed T cell responses much later, 278–366 days after infection. Data on specific T cell immunity after mpox infection are scarce, especially in HIV-positive patients [24], and we are not aware of any long-term data. Thus, the combined data by Agrati et al. [24] and our study indicate for the first time that T cell responses seem to be rather stable—even in HIV-positive patients—for almost a year after mpox infection. Interestingly, rhesus macaques vaccinated with a modified vaccinia virus Ankara (MVA) strain, containing genes from a pathogenic simian human immunodeficiency virus, developed long-term poxvirus-specific immunity [26], with even an increase in specific T cells within 34 weeks (238 days) after vaccination. As with vaccinia virus, T cells are likely to provide an important contribution to overall immunity to mpox infection [22]. However, according to challenge experiments in macaques, mpox-specific neutralizing antibodies may be even more important for viral control than T cells [27]. Recent data by Sammartino et al. [28] indicate that after infection and after vaccination, neutralizing antibodies increased as compared with naïve controls, and that a second dose of a poxvirus vaccine boosts the serological response to levels similar to that of the mpox-infected patients. Moreover, a case report described an asymptomatic mpox infection, in which neutralizing antibodies were detected on the 30th day after household contact with mpox [29]. Confirming the importance of neutralizing antibodies for the course of the disease, Zeggagh et al. described a man with a second clinical episode of mpox infection, who had abnormally low titers neutralizing antibodies [30]. Finally, studies with mousepox have shown that the interaction of T and B cells and antibodies is absolutely critical for recovery from a second challenge [25].

After vaccination with one dose of the MVA–BN smallpox vaccine, an effectiveness of 68–93% was reported; after two doses, it was 76–90% [31,32,33]. However, a recent large case-control study showed an effectiveness of only 36% against symptomatic mpox infection after a single vaccination, whereas the efficacy reached 66% after two vaccinations [34]. Symptoms may be less severe and less characteristic after prior vaccination [35,36]. In a large study from the U.S., comprising 5402 mpox-infected men aged 18–49 years, the mpox incidence was 14 times as high among unvaccinated men compared with those who had received a first vaccine dose ≥14 days earlier [37]. Nevertheless, despite vaccination, breakthrough infections can occur due to various factors, including only low levels of mpox-neutralizing antibodies, variations in the host immune response, exposure to high viral loads, the emergence of new variants of mpox, or a decrease in immune responses after vaccination [35,38,39,40,41,42,43].

Ideally, a positive orthopoxvirus-specific ELISpot result should indicate protective T cell immunity against mpox infection. However, it is challenging to set an optimal cut-off for positive T cell responses. The cut-off definition for our in house ELISpot assay was based on a previous study [13], with an adaptation to the number of PBMCs used per cell culture. Moreover, this cut-off was validated by comparing it with the negative controls of the current study (which all showed responses below the cut-off). Using our cut-off definition, vaccination against Poxviridae led to specific T cell immunity in at least 64% of HIV-positive patients. Thereby, an overall efficacy of 66% after two vaccinations [34] seems to fit well with our in vitro results. Of note, the strength of ELISpot responses is dependent on several variables, including the detection system and cell numbers, and thus cut-off definitions for ELISpot assays can substantially vary. For example, in a previous study by Koch et al. [44], which assessed MERS-CoV specific T cell immunity, the cut-off was set at 50 spots per million PBMCs, i.e., 15 spots per 300,000 PBMCs. When applying this rather stringent definition to our study, after vaccination, only up to 9% of the patients would display orthopoxvirus-specific IFN-γ or IL-2 ELISpot responses, which seems to underestimate the rate of protective T cell immunity. To assess whether the T cell responses to orthopoxviruses were rather low or high, the frequency of reactive T cells can be compared with SARS-CoV-2-specific T cells after two vaccinations, tested in immunocompromised patients also by our in house ELISpot assay. In recipients of hematopoietic stem cell transplants, we observed an even lower T cell response after vaccination against SARS-CoV-2 [45].

According to a recommendation by the Centers for Disease Control and Prevention (CDC), vaccination is an important tool in preventing the spread of mpox [46]. Two doses of the vaccine should be administered, which is considered as the best protection against severe illness, hospitalization, and death [38,46]. In addition to vaccination, the isolation of mpox patients and other measures to prevent the spread of infection, such as covering wounds, wearing medical masks, or using condoms, are further ways of reducing the infection rate [2]. These measures are especially important in the early weeks following vaccination and in low- or middle-income countries with limited access to vaccines, such as Brazil, where numbers of mpox-infected patients also declined [38,47,48,49].

In summary, infection and two vaccinations both induced long-term orthopoxvirus-specific T cell responses in HIV-positive patients, which should, in the majority of cases, protect against mpox infection, together with humoral immunity.

## 5. Conclusions

According to our data, vaccination against Poxviridae led to specific T cell responses in at least 64% of HIV-positive patients. After infection, T cell responses were detectable in all patients. The results of our cell culture experiments fit well with clinical observations, showing a substantial decrease in mpox infections after starting vaccination programs in risk groups. The duration of cellular and humoral immunity in HIV-positive patients has now to be assessed by follow-up analyses.

## Figures and Tables

**Table 1:** Characteristics of 43 HIV-positive patients tested for cellular immunity against mpox-orthopoxviruses.

Variable ^1^	Vaccinated (*n* = 33)	Infected (*n* = 10)
Age (years)	44 (24–63)	39 (25–59)
Interval second vaccination or infection—testing (days)	115 (51–247)	314 (278–366) ***
Absolute cell counts (cells/μL)		
CD3+	1710 (911–3290)	1895 (1560–2560)
CD4+	753 (335–1770)	950 (659–1490)
CD8+	821 (351–2150)	794 (540–1270)
Relative cell counts [%]		
CD3+	75.8 (41.3–84.2)	73.1 (59.8–83.0)
CD4+	34.1 (20.6–59.2)	34.9 (27.3–53.9)
CD8+	35.0 (9.3–55.4)	30.8 (20.7–39.6)
CD4/CD8 ratio	1.0 (0.4–29)	1.0 (0.7–2.6)
Statins (with/without)	3/30	1/9
Immunosuppressive therapy (with/without)	1/32	0/10

^1^ Data are given as median (range). Characteristics of the two patient groups were compared by Mann–Whitney test or Fisher’s exact test, as appropriate. Significant differences were found only for the interval between vaccination/infection and testing (*** *p* < 0.0001).

**Table 2:** Spearman correlation of orthopoxvirus-specific ELISpot responses in 10 patients after mpox infection.

ELISpot	Parameter	*r*	*p*
IFN-γ (1 μg/mL per peptide)	CD8+ T cells [%]	0.56	0.09
	CD4/CD8 ratio	−0.63	<0.05 *
IFN-γ (2 μg/mL per peptide)	CD8+ T cells [%]	0.57	0.09
	CD4/CD8 ratio	−0.57	0.08
IL-2 (1 μg/mL per peptide)	CD8+ T cells [%]	0.73	0.02 *
	CD4/CD8 ratio	−0.55	0.10
IL-2 (2 μg/mL per peptide)	CD8+ T cells [%]	0.69	0.03 *
	CD4/CD8 ratio	−0.65	0.04 *

* p < 0.05.

## Data Availability

The data presented in this study are available on request from the corresponding author. The data are not publicly available due to privacy restrictions.

## Supplementary Materials

The following supporting information is available online at https://www.mdpi.com/article/10.3390/vaccines12020131/s1, Figure S1: Optimization of an orthopoxvirus-specific ELISpot assay in 26 healthy controls.
