# Supplementary material for: T Cell Responses against Orthopoxviruses in HIV-Positive Patients
Source: Vaccines (Basel). 2024 Jan 27;12(2):131. doi: 10.3390/vaccines12020131 (PMC10891540; doi:10.3390/vaccines12020131)
Supplement: Supplementary file 1 [file vaccines-12-00131-s001.zip › vaccines-2756839-supplementary.pdf]

T cell responses against orthopoxviruses in HIV-positive patients

Supplementary data:

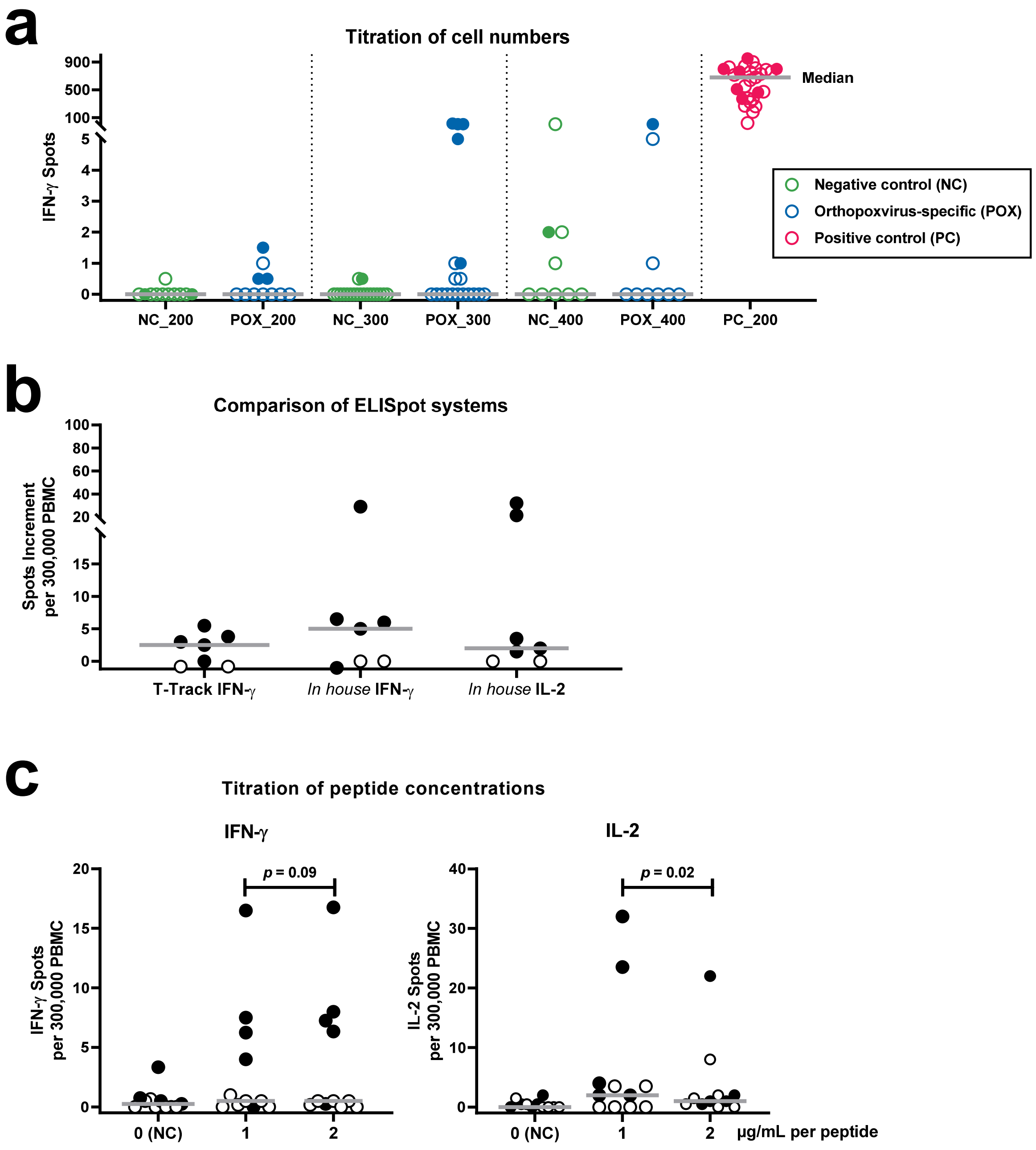

Figure S1. Optimization of an orthopoxvirus-specific ELISpot assay in 26 healthy controls. Panel (**a**) shows the titration of cell numbers (200,000 to 400,000 PBMC per culture, with 1 µg/mL of each peptide), panel (**b**) the comparison of three ELISpot systems (T-Track ELISpot kit vs. *in house* ELISpot assays, with 1 µg/mL of each peptide), and panel (**c**) the comparison of T cell responses after using two concentrations of a pool of 127 orthopoxvirus-specific peptides (1 and 2 µg/mL of each peptide). Open circles indicate that the controls were either non-vaccinated or received only one vaccination in early childhood. Filled circles mean that the controls were either vaccinated twice in early childhood or received one recent vaccination, after June 2022. Horizontal grey lines indicate median values. Cellular responses were either shown separately for the negative controls (NC) and orthopoxvirus-specific response (**a**,**c**) or as incremental value, i.e. specific response minus NC (**b**).
